# Frailty is associated with worse cognition and depressive symptoms: an ELSI‐Brazil study

**DOI:** 10.1002/alz70858_104902

**Published:** 2025-12-25

**Authors:** Joao Senger, Joana Emilia Senger, Wyllians Vendramini Borelli, Eduardo R. Zimmer

**Affiliations:** ^1^ Instituto Moriguchi, Veranópolis, Rio Grande do Sul, Brazil; ^2^ Universidade Federal do Rio Grande do Sul, Porto Alegre, RS, Brazil; ^3^ Universidade Federal do Rio Grande do Sul, Porto Alegre, Rio Grande do Sul, Brazil; ^4^ Centro de Memória, Hospital Moinhos de Vento, Porto Alegre, RS, Brazil; ^5^ Federal University of Rio Grande do Sul (UFRGS), Porto Alegre, RS, Brazil; ^6^ McGill Centre for Studies in Aging, Montreal, QC, Canada

## Abstract

**Background:**

Frailty syndrome is an older adult condition that gradually loses physical abilities and capacity to recover. This situation makes their vulnerability to negative health outcomes as falls, fractures, and dementia. Therefore, the aim of this study is to understand the association between frailty, cognitive decline, and mental health factors related to life satisfaction.

**Method:**

This analysis utilized data from the longitudinal study on the health of older Brazilian adults (ELSI‐Brazil), this is an epidemiological study that employs complex sampling methods to obtain a representative sample of the entire Brazilian territory. Sociodemographic data, immediate and delayed memory scores and semantic fluency scores, were gathered to calculate a cognitive Z‐Score. Participants were asked, “In the last month, how many days was your mental health not good, such as feeling depressed, stressed, or having emotional problems?”, a life satisfaction score and the “Do you feel excluded?” were included to correlate with others data. The frailty score was based on the Cardiovascular Health Study, calculated factors (ranging from 0 to 5). Predictors of frailty were assessed using linear regression conducted in R (survey package).

**Result:**

The study included 7,564 individuals of both sexes (mean age: 62.4±9.3 years), with an average of 5.6 (±4.4) years of education. Significant associations were identified between frailty and cognitive scores (beta?, *p* <0.001). Individuals presenting frailty also demonstrated higher frequency of depressive symptoms than controls (9.4±12.2 vs. 4.9±9.4, *p* <0.0001). Differences in life satisfaction were also significant between groups (X±SD vs. Y±SD, *p* <0.0001). A discrepancy emerged in the response “always” regarding feelings of exclusion between frail and non‐frail participants (29% vs. 15%, *p* <0.0001), even after adjusting for sex, age, education, and financial status.

**Conclusion:**

These findings highlight a link between frailty and cognitive function, along with mental health indicators such as depression and feelings of exclusion in older Brazilian adults. This suggests that, in addition to cognitive decline, social isolation may influence frailty. The results show the importance of inclusivity to enhance the quality of life for frail individuals and potentially prevent dementia.

